# Coordinating health promotion in a federal state over the course of 30 years: a case report from Switzerland

**DOI:** 10.15171/hpp.2019.45

**Published:** 2019-10-24

**Authors:** Thomas Mattig

**Affiliations:** Institute of Global Health, University of Geneva, Geneva; Health Promotion Switzerland, Bern

**Keywords:** Health policy, Health promotion, Prevention

## Abstract

In a federal state such as Switzerland, the 26 cantons enjoy wide autonomy in all policy areas, including health policy. In the late 1980s, after the signing of the Ottawa Charter for health promotion, the Swiss Confederation and the cantons decided to create a foundation whose goal it was to initiate, coordinate and evaluate health promotion and disease prevention activities throughout the country. Thirty years later, the many stakeholders in the field have accepted health Promotion Switzerland as a key actor. The foundation was able to successfully initiate and coordinate projects in such priority areas as "Healthy Body Weight," "Mental Health –Stress" and "Strengthening Health Promotion & Prevention." But several challenges remain, for example, chronic noncommunicable diseases, an aging population, mental disorders, prevention in healthcare—challenges the foundation will have to face and to which it is expected to provide solutions.

## Introduction

In 1848, Switzerland became a federation of 26 sovereign cantons. The cantons have enjoyed wide autonomy in all policy areas, including health policy, ever since.^1^ Indeed, each canton has its own legal framework to regulate healthcare (e.g., inpatient care planning), disease prevention (e.g., implementation of screening programs), health promotion (e.g., development of healthy behavior projects at schools) as well as specific regulations regarding the allocation of health resources.^2^ To some extent, the observed heterogeneous cantonal health policies reflect variations among the cantons in terms of available resources; demographic population structures (urban versus rural); religious, cultural and linguistic backgrounds (e.g., four official national languages); or geographical characteristics (cities in the flatlands versus mountain villages) in a country with an overall population of roughly 8 million.^3^ This led some experts to describe the Swiss health system as having “evolved in largely fragmented and uncoordinated fashion,”^1^ all the more so in that there are many different stakeholders, be they public (such as municipalities) or private (such as health leagues, local health associations and insurance companies).^4^

Even though the cantons still have the core responsibilities in health policy matters, the federal government’s role has increased over the years, especially through the adoption of federal laws on the insurance coverage of accidents, invalidity and disease.^5^ Yet there is no federal health act per se that would set an overall framework for health promotion, disease prevention and care.^6^

This case report describes the development, activities and achievements over the 30 years of the foundation’s existence as well as its present and future challenges.

## Case Report

### The foundation’s early years (1989–1993)

In the late 1980s, after the signing of the Ottawa Charter for Health Promotion and following the refusal of the cantons and the business community to support a federal bill on prevention, the Swiss Confederation and the cantons, in association with the “Swiss Association of Private Health and Accident Insurers” and the “Concordat of Swiss health insurers,” decided to create the Swiss Health Promotion Foundation with CHF 1.6 million in initial funding (CHF = Swiss francs; one Swiss franc being roughly equivalent to 1 US dollar). Its original mandate focused on the conception of a national smoking cessation campaign to be implemented in collaboration with the Swiss Cancer League and the Tobacco Control Fund and with the support of the Federal Office of Public Health.^7^ At the same time, the foundation compiled an inventory of the organizations active in health promotion and disease prevention throughout the country and started to sponsor grassroots health promotion and disease prevention programs in the cantons on an annual basis. It also initiated, in collaboration with academic institutions, the development of documentation centers specializing in scientific and professional literature on health promotion and disease prevention. Eventually it developed a collaborative network of health professionals and policy decision-makers from the different cantons and various health organizations.

From the start, the Ottawa Charter’s health promotion framework has served as a conceptual basis for the foundation’s strategic orientation, namely developing personal skills, creating supportive environments, strengthening community action, reorienting health services and building healthy public policy.^8^

### Consolidating the foundation (1994–2006)

In 1994, five years after its establishment, the existence of the foundation was enshrined in the newly adopted Health Insurance Act,^9^ which stipulates in its Article 19 that “insurers promote disease prevention; together with the cantons, they operate an institution that initiates, coordinates and evaluates measures to promote health and prevent diseases.” Article 20 sets the framework for financing the foundation: “An annual contribution for general disease prevention is collected from each person covered by compulsory insurance under this Act.” As of 1998, the contribution amounted to CHF 2.40 per person per year, which made for an almost tenfold increase of the foundation’s annual budget compared to the previous years. By 2018, the contribution had doubled, to CHF 4.80 per person per year.

This budget increase enabled the foundation to fund a wide range of projects designed and implemented by disease prevention and health promotion stakeholders across the country. By way of example, the topics supported by the foundation in 1998, the year of the initial budget increase, covered addiction, nutrition, women’s health, violence prevention, health promotion for migrants, promotion of youth health. In the five-year span between 1998 and 2002, 465 projects were funded (out of a total of 1566 submitted proposals).

The increased budget also enabled the foundation to identify priority areas for large-scale interventions to be progressively developed and implemented in coordination with partner institutions in the cantons, taking into account the fact that those priority areas evolved over the course of more than a decade: these include *Priority program 1*: Healthy behavior (physical exercise, nutrition, relaxation); *Priority program 2*: Health and Work; *Priority program 3*: Adolescents and young adults. Table 1lists some examples of projects supported, funded and evaluated by the foundation in these three priority areas, where the foundation ensured the overall coordination.

At the same time, the foundation developed a strong communication and advocacy strategy to raise awareness among the population and policymakers of the benefits of disease prevention and health promotion.

### The 2007–2018 strategic plan and its implementation

The foundation’s 2008–2018 strategic plan is summarized in Table 2.

The long-term strategy was based on two priority areas “Healthy Body Weight” and “Mental Health – Stress” and the overarching strategic goal “Strengthening Health Promotion & Prevention,” to be implemented over two four-year periods, followed by a period of further development between 2016 and 2018.^10^

The priority area “Healthy Body Weight” focused on children and adolescents in school settings. The program was implemented through the Cantonal Action Programs (CAPs) in the cantons committed to promoting healthy eating and physical exercise while adopting four approaches: implementing projects, adapting policies, networking and informing the population. The foundation supported the cantons by making available evidence-based knowledge and best practices as well as communication materials, by funding activities, and through networking and coordination.

In the area of “Mental Health – Stress,” activities were centered on workplace settings, with the aim to help workers and employees better control their stress and for employers to create working condition conducive to that aim. The foundation developed a set of workplace health management (WHM) tools, in particular the S-Tool, an online survey instrument that supports companies in uncovering stressful working conditions. The foundation also created the label *Friendly Work Space*,^11^ which is awarded to companies that fulfill a series of criteria in terms of improving working conditions that can reduce workers’ or employees’ stress levels and lead to an overall improvement in their health.

“Strengthening Health Promotion & Prevention” focused on the population and on policymakers, with the goal of raising their awareness of the importance of prevention and health promotion. It also focused on developing and managing a network of various key stakeholders in the field of public health through a series of activities—for example, an annual conference on specific health promotion topics, or workshops allowing health promotion professionals to exchange their experiences, doubts, questions, materials, etc. In addition, innovative projects on topics outside the defined priority areas were initiated and supported, and specific project management tools were developed.

### The 2019–2024 Strategy and its priorities

The cornerstones of the foundation’s 2019–2024 Strategy are outlined in Table 3. In addition to its “traditional” activities, i.e., lobbying for health promotion and disease prevention as well as extending and coordinating the CAPs and supporting WHM, the foundation has been mandated with specific new foci:

• *Noncommunicable diseases,*which affect 2.2 million people in Switzerland and are responsible for 60% of premature deaths and 80% of overall healthcare costs^12^:

♦ The foundation specifically supports *prevention in healthcare*, with the aim of strengthening prevention across the entire supply chain to promote quality of life and patient autonomy and to reduce the need for treatment. Table 4 lists the projects selected in 2018 and funded for the years 2019–2020 and 2019–2022, respectively.^13^

• *The elderly*,an especially vulnerable population group from a health point of view. Indeed, 25% of those aged between 65 and 79 suffer from multiple diseases; in the age 80-plus age group, which has an especially high risk of falls, 41% suffer from multiple diseases; and among those over 75, around 30% suffer from loneliness.^14^

♦ Table 5 shows the main axes of prevention aimed at the elderly and specific activities being either initiated or coordinated by the foundation.^15^

• *Mental health*, a priority public health problem, given that 18% of people living in Switzerland report suffering from psychological problems (25% of whom consider their problems major), primarily mood disorders and depression.^16,17^ On average, these reported disorders are more frequent in women than in men, yet suicide rates are three times higher in men than in women, with a global rate of 13/10^5^(European average: 11/10^5^). The burden of disease due to mental disorders represents 13.6% of the total number of disability-adjusted life years—third position behind cancers (16.0%) and musculoskeletal disorders (14.1%). The situation is further compounded by the fact that people with mental health problems are frequently stigmatized and discriminated against.^18^

 ♦ The foundation coordinates national campaigns to raise public awareness of mental health in order to reduce discrimination and stigmatization of people suffering from mental disorders and their families. It also publishes factsheets and guidelines as well as reviews of the literature and empiric data for health professionals, policymakers and laypersons. Examples are provided in Table 6.

Mental health is also closely correlated to stress in the workplace, with Siegrist^19^ reporting an odds ratio of about 1.8 of depression among workers exposed to high demand and low control at work or who made significant efforts while receiving low recognition, all of which are stress situations that are well covered in the literature.^20^ Recently collected data in Switzerland measuring stress at work via the Job Stress Index indicate that roughly one-quarter of the working population are stressed at work, with an equal number feeling exhausted.^21^

♦ The foundation supports companies in their efforts to prevent stress for their employees as well as burnout and depression related to constraints and conflicts in the workplace.

## Discussion

### Basic considerations

In Switzerland, three public bodies share the responsibility of defining health policy: the federal authorities, the cantons and the municipalities—bodies that have to take into account the interests and demands of a multitude of stakeholders, such as national and cantonal health leagues, insurance companies, pharmaceutical companies, professional and consumer associations, etc. This implies that decision-making in terms of public health involves negotiations among all parties and, ultimately, the establishment of a consensus (which is sometimes the lowest common denominator that could be agreed upon). Since it’s the citizens who ultimately decide in a direct democracy such as Switzerland, such consensus is never final but is always being reviewed and redefined.

That spirit of consensus has driven the approach chosen by Health Promotion Switzerland since its creation thirty years ago, i.e., participative leadership integrating the main stakeholders in the field. This allowed the foundation to gradually overcome suspicions from various stakeholders, especially the health leagues, which have themselves been active in health promotion and disease prevention for decades. As has been observed in other Swiss public health contexts,^15^ such a “progressive process respected the cantonal heterogeneities”^15^ and might have been key to the foundation’s long-term acceptance.

### Some evaluation data

An external evaluation^11^ of the 2007–2016 period, as well as internal evaluations,^22^ reports tangible successes in the three priority areas but also mentions some challenges to be addressed in the future. Summing up:

- *“Healthy Body Weight”:*Focused on children and adolescents, the program was carried out through the CAPs and implemented in 20 cantons (out of 26), which allowed it to reach an estimated number of more than 600 000 young people through specific modules/activities on healthier diet and/or increased physical exercise. The effort seems to be paying off, as the evaluation data collected between 2005/2006 and 2014/2015 reports a decrease (from 19.5% to 17.3%) in the share of overweight or obese children and adolescents. Yet even though the literature emphasizes the effectiveness of this dual diet/physical exercise approach,^23,24^ the complexity of the issue suggests we should be cautious about attributing this decrease to CAPs alone. Nevertheless, the CAPs also contributed to the strengthening and functioning of the health promotion network. Other activities, such as the national campaigns promoting physical mobility (the so-called “slowUp” days), have mobilized tens of thousands of participants every year, more recently even several hundred thousand (400 000 in 2016).
- *“Mental Health – Stress”:* The implementation of the strategy in this priority area is characterized by the focus on stress reduction on individual as well as well organizational levels: valuable products were developed, such as the Friendly Work Space label and the S-Tool. In fact, over the years, the WHM project enabled the foundation to cooperate with as many as 75 companies, representing almost a quarter of a million employees, to better understand the conditions likely to foster or remedy stress in workers. The project also permitted the annual collection, from a representative sample of the Swiss working population, of data on the imbalance between work constraints and resources (the Job Stress Index, an indicator of stress-inducing conditions in the workplace), workers’ perceived level of exhaustion and the resulting potential economic impact: as many as 25% of those surveyed feel very stressed at work, with the same number feeling emotionally exhausted.Beneficial effects of the program are likely, since the literature suggests that even “a brief workplace stress management intervention can produce clinically significant reductions in blood pressure and improve emotional health among hypertensive employees,”^25^ which was a long-contested claim.^26^
- *“Strengthening Health Promotion*&*Prevention”:* A 2018 evaluation report^10^ states that the strategy convinced with successful networking activities in and between cantons as well as with associations and organizations active in the promotion of health and the prevention of diseases. In fact, the foundation has been able to establish close collaborations with numerous partners over the years, either through financial support, as initiator or coordinator of local, regional or national projects, or by providing know-how, tools and guidelines in the field of prevention and health promotion to the various stakeholders. It has repeatedly been reported in the literature that such partnerships and cooperations/collaborations contribute to the success of intervention programs.^27-29^

### Present and future challenges

At present the foundation has attained its cruising speed, and a solid financial footing will allow it to face present and future challenges, e.g.:

- Noncommunicable diseases: One of the long-term challenges the Swiss health system is facing are chronic noncommunicable diseases (NCDs).^30^ Federal authorities have recently decided to tackle the problem more aggressively, and initiated a national NCD-prevention strategy that focuses on five health problems: cardiovascular diseases, diabetes, cancer, chronic respiratory diseases and musculoskeletal disorders.^31^ Health Promotion Switzerland closely collaborated with the federal and cantonal authorities in developing the action plan for the NCD strategy, which comprises three main measures: population-based health promotion and prevention, prevention in healthcare and prevention in industry and the workplace, in addition to such cross-cutting measures as facilitating the funding of specific risk-factor projects, connecting the various stakeholders through existing and new networks, monitoring trends in relevant NCD indicators and raising NCD awareness among the population.^32^
- An aging population: The health problems associated with an aging population make it a priority for the foundation to provide older people with a better quality of life and more autonomy in the long term. Four areas will be favored, namely the promotion of physical exercise, healthy eating, mental health and the prevention of falls. Indeed, among people over 65 living at home, 49% suffer from at least one chronic disease, whereas 25.2% of those aged 65 to 79 report suffering from several chronic diseases. For people in their 80s, that percentage rises to 41.3%, and one-third of them are at high risk of falling within the year. Furthermore, between 15% and 25% of those over 65 suffer from at least one mental disorder.^33^

Programs targeting the elderly will be integrated into the CAPs, taking into account the experiences of the VIA project.^15^

- Mental health: Mental health is another key challenge the Swiss health system is confronted with. Its importance for Switzerland has been stressed in a recent report by the federal and cantonal authorities and Health Promotion Switzerland.^16^ The report recommends urgent action, notably in terms of raising awareness of the importance of mental health among the population and to avoid stigmatizing people who suffer from mental disorders. It also recommends reinforcing health promotion and prevention in the area of mental health as well as early screening of mental disorders. Indeed, public health issues related to mental health are important (every year 38.2% of the European population suffer from mental health problems; in terms of disability, these diseases represent 26.6% of the total disease burden).^34^ In Switzerland, the costs related to mental health disorders are estimated to account for 16% of overall health costs).^35^
- Disease prevention and health promotion in healthcare: In line with the federal NCD Strategy, the foundation will focus on developing and strengthening prevention in healthcare. In addition to primary prevention measures, the focus will be on the early detection of diseases and their risk factors. Backed by international recommendations,^36,37^ empowering patients, strengthening their resilience and improving their quality of life are additional objectives, as is strengthening the skills of health professionals in early disease detection and health promotion.

## Conclusion

Over the years, the foundation has become a key actor in the field of health promotion and disease prevention, widely accepted by the health authorities at local, regional and national levels as well as by the many stakeholders in the field. But reaching this point required a lengthy process aided by the partnership approach the foundation adopted from the beginning and by the generous funding enshrined in law. Yet the challenges of the coming years will be numerous—an aging population, the impact of environmental pollution^38^ on health or the potential utility of IT applications as health promotion tools^39^—and adaptability a must.

## Ethical approval

Not relevant.

## Competing interests

None.

## Funding

No external funding.

## Authors’ contributions

Conception, writing and corrections by the author.

## Acknowledgments

Thanks to the administrative staff of the Foundation for providing the Annual Reports of the Foundation.

**Table 1:** Examples of projects supported by the foundation in its priority areas during the period 1998–2006

**Priority area**	**Name of the project**	**Headed by**	**Subject**	**Duration**	**Totalbudget** ^a^ **(% funded by HPS)**
Healthy Behavior	Go Hop	Federal Office for Sports	National program to promote physical exercise	2003–2005	CHF 5 100 000 (29%)
	slowUp	SwitzerlandMobility Foundation	Promotion of soft mobility on roads closed to traffic on specific days	2003–2005	CHF 3 360 000 (16%)
	5 a Day	Swiss Cancer League	Promotion of the consumption of 5 servings of fruits and vegetables per day	2004–2005	CHF 320 900 (31%)
Health and Work	Health Promoting Hospitals	ZH-Hospital Association	Improving the health of patients, staff and communities by integrating health promotion across the organization	2003–2004	CHF 454 000 (24%)
	Intercantonal Health Promotion in the Enterprise	Cantons Zug, Zurich, Aargau, St. Gallen, Thurgau	Implementing a WHM strategy to improve employee health	2005–2006	CHF 889 000 (49%)
Adolescents and Young Adults	Parents and Schools Strengthen Children	University of Applied Sciences and Arts Northwestern Switzerland	Promotion of health and prevention of stress, aggression and addiction in school settings	2005–2006	CHF 965 459 (14%)
	Health-Promoting Schools	Radix Foundation	Development of a national network of schools committed to health promotion	2005–2006	CHF 2 833 500 (43%)

Abbreviation: WHM, workplace health management; HPS, Health Promotion Switzerland. ^a^CHF = Swiss francs.

**Table 2:** The foundation’s 2007–2018 strategic plan

**Healthy Body Weight**	Preparation phase	Disseminate the Action Programs in the cantons				Consolidate the Action Programs in the cantons				Further develop the content of the Action Programs	
		Cantonal Action Programs (CAPs) Approach: modular programs, networking, lobbying policymakers, informing the population Topics: healthy food, physical exercise Priority target population: children and adolescents Settings: schools/kindergartens								Extension of Cantonal Action Programs to mental health the elderly	
		National campaigns promoting physical mobility – “slowUp” National campaigns against sugary beverages – “Drink Water”									
**Mental Health – Stress**		Promote the development of stress management skills among employees				Disseminate the workplace health management project (WHM)				Raise awareness of the WHM approach in various professional sectors	
		Develop and disseminate the S-Tool/Job Stress Index				Create and award the Friendly Work Space (FWS) Label				Communicate regarding the FWS label in order to increase the interest of companies	
**Strengthen Health Promotion & Prevention**		Strengthen health promotion and disease prevention								Integrate the federal NCD Strategy	
		Health promotion and communication campaigns to raise health promotion and prevention awareness among the population and policymakers Consolidate the health promotion network with the various stakeholders Support innovative health promotion projects								Support health promotion and prevention in healthcare Focus CAPs on NCDs	
		Develop communication materials, evidence-based guidelines, best practice recommendations, project management tools									
**Time line**	2007	2008	2009	2010	2011	2012	2013	2014	2015	2016	2017

**Table 3:** The foundation’s 2019–2024 strategic objectives

**Statutory mandate**	**Areas of intervention**		
	**Cantonal Action Programs**	**Workplace Health Management**	**Prevention in Healthcare**
**Initiate**	*Objective 1* The cantons champion mental health, a balanced diet and sufficient physical exercise for children, adolescents and older people.	*Objective 2* Employers effectively champion the mental health of their employees and systematic workplace health management.	*Objective 3* The potential of prevention in healthcare to combat NCDs, mental illness and addiction has been demonstrated and the dissemination of effective projects by stakeholders in healthcare has been organized.
**Coordinate**	*Objective 4* The selected mental health promotion campaigns have been further developed and coordinated.		
	*Objective 5* The stakeholder in health promotion and prevention are networked, learn from one another and work together effectively and efficiently.		
**Evaluate**	*Objective 6* The effectiveness of measures by Health Promotion Switzerland has been reviewed and demonstrated to policymakers, the public and stakeholders in health promotion and prevention.		

**Table 4:** Health promotion projects in healthcare selected by the foundation in 2018

**Name of the project**	**Subject**	**Headed by**	**Duration (month)**	**HPS contribution** **(% of total budget)**
A Better Life with COPD	National dissemination of a self-management coaching targeting COPD patients to promote health literacy and a better quality of life	Swiss Lung League	48	CHF 400 000 (11.5%)
dAS-Tool	Development of a digital alcohol self-management tool via chatbot to prevent relapses	Health Bern Foundation	24	CHF 199 890 (35%)
INTERMED +	Secondary prevention targeting patients of GPs presenting risk factors for NCDs	General practice Neuchâtel	24	CHF 200 000 (91%)
I Move for My Health	Promote services to develop healthier lifestyles targeting people with NCDs	Diabetes Association Vaud	24	CHF 200 000 (74%)
No Secrets at Home	Regional dissemination of a project on participatory prevention of domestic violence against children	National Coalition Building Institute	48	CHF 400 000 (36%)
KOMPASS	Acquisition of competences by those affected by musculoskeletal diseases through coaching by medical practitioners	Swiss Rheumatism League	24	CHF 200 000 (58%)
Step by Step 2	Resumption of physical exercise for sedentary persons at risk of NCDs	Health Promotion Vaud	48	CHF 1 999 000 (100%)
Prevention of Diabetes	Development of a network of professionals and implementation of early treatment programs of diabetes	The two Diabetes Associations Valais	48	CHF 2 000 000 (74%)
Psychiatric Joint Crisis Plan	Aims to improve the quality of life of psychiatric patients	Health Network Vaud	24	CHF 197 000 (59%)
Self-check Health-Competent Organization	With the help of a self-assessment tool, primary care providers are to be empowered to strengthen the health literacy of patients	Health Department Zurich	24	CHF 200 000 (64%)
SomPsyNet	Early screening and rapid treatment of psychosocial stress and psychiatric disorders of patients in acute somatic hospitals	Health Department Basel-Stadt	48	CHF 2 000 000 (100%)
Strong Family	Focuses on the early detection and intervention targeting families with overweight infants/children	Association Obesity in Childhood and Adolescence	24	CHF 200 000 (94%)
Fall Prevention	Professionals are to be enabled to recognize patients at risk of falling and to provide appropriate preventive measures	Health Department St. Gallen	48	CHF 2 000 000 (49%)
Win Back Control	Development and dissemination of a Web-based self-help platform for the reduction of problematic or pathological gambling	Institute for Addiction Research	24	CHF 199 776 (51%)

**Table 5:** Examples of integrated health promotion activities targeting the elderly coordinated by the foundation

**Field of coordination**	**Specific activities**
Evidence-based interventions	Establishing and disseminating an evidence-based database on health-promoting activities targeting the elderly
Promotion of physical exercise	Developing and coordinating a “municipality alliance on promoting physical exercise among the elderly” network Holding specificworkshops for professionals on how to promote physical exercise among older people Organizing round tables for the elderly with sports representatives on physical exercise and sports in various cantons
Fall prevention	Lobbying in favor of concrete measures to reduce the risk of falls at home Conceiving and implementing national campaigns promoting physical exercise among the elderly Conceiving and disseminating information (e.g., leaflets containing descriptions of various exercises) targeting the elderly
Mental health	Initiating studies on social exclusion and loneliness as a mental health risk factor Conceiving and coordinating a multi-site program promoting social integration for the elderly Promoting structural measures aimed at decreasing the discrimination and stigmatization of people suffering from mental disorders and their families
Integration of general practitioners	Planning and implementing an information campaign targeting general practitioners on the usefulness of health-promoting activities for the elderly Funding of health-promoting projects targeting chronic patients of general practitioners

**Table 6:** Examples of publications by the foundation as a contribution to the promotion of mental health

**Year of publication**	**Title and content**	**Target population**
2019	*Social resources*. The promotion of social resources as an important contribution to mental health and a good quality of life	Community health personnel and social workers
2019	*Health of volunteer community caregivers.* A guide for promoting the mental health of community caregivers (relatives, friends, neighbors) of the elderly	Community caregivers
2019	*Promoting mental health in early childhood.*Recommendations for health and social service professionals	Community health personnel and social workers
2019	*Self-efficacy.*Develop among children and adolescents the conviction of having the necessary skills to effectively perform desired actions (with a focus on health-protecting behaviors and risk factors)	Health personnel, teachers
2017	*Healthy body image for young people in Switzerland.*	Teachers, parents
2017	*Sexual and gender minorities in health promotion and prevention.*Specifics to take into consideration in health promotion and prevention targeting LGBTI populations	Community health personnel, teachers, adolescents
2016	*Mental health over the course of life.*	Community health personnel
